# PD-L1 expression in equine malignant melanoma and functional effects of PD-L1 blockade

**DOI:** 10.1371/journal.pone.0234218

**Published:** 2020-11-20

**Authors:** Otgontuya Ganbaatar, Satoru Konnai, Tomohiro Okagawa, Yutaro Nojima, Naoya Maekawa, Erina Minato, Atsushi Kobayashi, Ryo Ando, Nobuya Sasaki, Daisuke Miyakoshi, Osamu Ichii, Yukinari Kato, Yasuhiko Suzuki, Shiro Murata, Kazuhiko Ohashi

**Affiliations:** 1 Department of Disease Control, Faculty of Veterinary Medicine, Hokkaido University, Sapporo, Japan; 2 Department of Advanced Pharmaceutics, Faculty of Veterinary Medicine, Hokkaido University, Sapporo, Japan; 3 Department of Veterinary Clinical Medicine, Faculty of Veterinary Medicine, Hokkaido University, Sapporo, Japan; 4 Laboratory of Veterinary Pathology, School of Veterinary Medicine, Kitasato University, Towada, Japan; 5 Laboratory of Laboratory Animal Science and Medicine, School of Veterinary Medicine, Kitasato University, Towada, Japan; 6 Mitsuishi Animal Medical Center, NOSAI Minami, Shinhidaka, Japan; 7 Department of Basic Veterinary Sciences, Faculty of Veterinary Medicine, Hokkaido University, Sapporo, Japan; 8 Department of Antibody Drug Development, Tohoku University Graduate School of Medicine, Sendai, Japan; 9 New Industry Creation Hatchery Center, Tohoku University, Sendai, Japan; 10 Division of Bioresources, Research Center for Zoonosis Control, Hokkaido University, Sapporo, Japan; University of Ulsan College of Medicine, REPUBLIC OF KOREA

## Abstract

Programmed death-1 (PD-1) is an immunoinhibitory receptor expressed on lymphocytes. Interaction of PD-1 with its ligand PD-ligand 1 (PD-L1) delivers inhibitory signals and impairs proliferation, cytokine production, and cytotoxicity of T cells. In our previous studies, we have developed anti-bovine PD-L1 monoclonal antibodies (mAbs) and reported that the PD-1/PD-L1 pathway was closely associated with T-cell exhaustion and disease progression in bovine chronic infections and canine tumors. Furthermore, we found that blocking antibodies that target PD-1 and PD-L1 restore T-cell functions and could be used in immunotherapy in cattle and dogs. However, the immunological role of the PD-1/PD-L1 pathway for chronic equine diseases, including tumors, remains unclear. In this study, we identified cDNA sequences of equine PD-1 (EqPD-1) and PD-L1 (EqPD-L1) and investigated the role of anti-bovine PD-L1 mAbs against EqPD-L1 using *in vitro* assays. In addition, we evaluated the expression of PD-L1 in tumor tissues of equine malignant melanoma (EMM). The amino acid sequences of EqPD-1 and EqPD-L1 share a considerable identity and similarity with homologs from non-primate species. Two clones of the anti-bovine PD-L1 mAbs recognized EqPD-L1 in flow cytometry, and one of these cross-reactive mAbs blocked the binding of equine PD-1/PD-L1. Of note, immunohistochemistry confirmed the PD-L1 expression in EMM tumor tissues. A cultivation assay revealed that PD-L1 blockade enhanced the production of Th1 cytokines in equine immune cells. These findings showed that our anti-PD-L1 mAbs would be useful for analyzing the equine PD-1/PD-L1 pathway. Further research is warranted to discover the immunological role of PD-1/PD-L1 in chronic equine diseases and elucidate a future application in immunotherapy for horses.

## Introduction

Programmed death-1 (PD-1) is an immunoinhibitory receptor, which is expressed on activated and exhausted T cells [1–3]. Programmed death ligand 1 (PD-L1), also called CD274, is the ligand of PD-1 expressed on immune cells, including antigen-presenting cells, and tumor cells [1, 4, 5]. The interaction of PD-1 and PD-L1 suppresses the activation signal mediated by T-cell receptors and inhibits effector functions of T cells, including cytokine production and cell proliferation [1–4]. This pathway is invaluable for regulating excessive immune responses [6–8]; however, in cancers, tumor cells utilize the suppression of T cells mediated by PD-1/PD-L1 to circumvent anti-tumor immune responses [9–11]. In human medicine, the blocking antibodies targeting PD-1 or PD-L1 have been leveraged for treatment of various types of cancers and resulted in remarkable outcomes with 20%–90% response rates in multiple clinical trials [12–15].

Equine malignant melanoma (EMM) is a common neoplasm among aged gray horses, resulting in dermal tumors at multiple sites [16]. A previous study reported that approximately 80% of aged gray horses developed dermal melanoma and speculated that all gray horses would develop this tumor as they reach old age [17]. Although cellular immune response is critical for eradicating melanoma, but several mechanisms have been suggested to limit anti-tumor immunity in EMM based on the findings for human malignant melanoma [18]. However, no studies are available on immune evasion mechanisms in EMM, and immune exhaustion mediated by PD-1 and PD-L1 has not been investigated in horses.

In our previous research, we established anti-bovine PD-L1 monoclonal antibodies (mAbs) [19]. We found that PD-1 and PD-L1 play crucial roles in immune exhaustion and disease progression in bovine chronic infections [20–24] and in canine cancers including malignant melanoma [25, 26]. Importantly, we noted that the PD-1/PD-L1 blockade enhances T-cell responses in cattle and dogs [20–26] and exhibits therapeutic effects in bovine chronic infections and canine malignant melanoma [27–31].

Until now, no information was available on cDNA sequences, expression, and function of PD-1/PD-L1 in horses. Furthermore, the role of the PD-1/PD-L1 pathway in EMM remained unclear. Based on the findings of our previous studies, we hypothesized that PD-1 and PD-L1 may provide potential targets for immunotherapy against EMM. Therefore, in this study, we identified cDNA sequences of equine PD-1 (EqPD-1) and PD-L1 (EqPD-L1), evaluated the blocking effects of our anti-bovine PD-L1 mAbs against EqPD-L1, and confirmed the expression of PD-L1 on EMM.

## Materials and methods

### Horse blood samples and cell preparation

Heparinized blood samples were collected from Thoroughbred horses in farms and veterinary hospitals in Hokkaido, Japan. Peripheral blood mononuclear cells (PBMCs) were purified using density gradient centrifugation on Percoll (GE Healthcare, Little Chalfont, UK), washed three times with phosphate-buffered saline (PBS), and suspended in PBS. All experimental procedures were conducted following approval from the local committee for animal studies according to the Hokkaido University (20–0093). Informed consent was obtained from all owners.

### Cloning of cDNA encoding of equine PD-1 and PD-L1

Equine PBMCs (4 × 10^6^ cells) were cultivated with 20 ng/mL of phorbol 12-myristate acetate (PMA; Sigma–Aldrich, St. Louis, MO, USA) and 1 μg/mL of ionomycin (Sigma–Aldrich) in RPMI 1640 medium (Sigma–Aldrich) supplemented with 10% heat-inactivated fetal bovine serum (FBS) (Thermo Fisher Scientific, Waltham, MA, USA), 2 mM of L-glutamine, 100 U/mL of penicillin, and 100 μg/mL of streptomycin (Thermo Fisher Scientific) at 37°C with 5% CO_2_ for 24 h.

Total RNA was isolated from cultivated PBMCs using of TRI Reagent (Molecular Research Center, Cincinnati, OH, USA) in accordance with the manufacturer’s instructions. Residual DNA was removed from RNA samples by treatment with Deoxyribonuclease I (Thermo Fisher Scientific). cDNA was synthesized from 1 μg of total RNA with PrimeScript Reverse Transcriptase (Takara Bio, Otsu, Japan) and oligo(dT) primer in accordance with the manufacturer’s instructions.

Gene-specific primers were designed to amplify EqPD-1 and EqPD-L1 genes, based on the sequences from horses available on GenBank (XM_005610777 and XM_001492842) (S1 Table). EqPD-1 and EqPD-L1 cDNAs were amplified by PCR using TaKaRa Ex Taq (Takara Bio) and specific primers (S1 Table). The PCR products were purified using a FastGene Gel/PCR Extraction Kit (Nippon Genetics, Tokyo, Japan) and cloned into the pGEM-T Easy Vector (Promega, Madison, WI, USA). They were transferred into *Escherichia coli* HST08 Premium Competent Cells (Takara Bio) and plated onto LB agar plates (Sigma–Aldrich) containing X-gal (Takara Bio) and ampicillin (Sigma–Aldrich). The purified plasmid clones were sequenced using a GenomeLab GeXP Genetic Analysis System (SCIEX, Framingham, MA, USA). The established sequences were aligned and an unrooted neighbor-joining tree was constructed using MEGA software program version 7.0 [32].

### Preparation of EqPD-1- and EqPD-L1-expressing cells

cDNAs encoding EqPD-1 and EqPD-L1 were amplified by PCR using gene-specific primers with restriction enzyme cleavage sites (S1 Table) and subcloned into the multicloning site of pEGFP-N2 (Clontech, Palo Alto, CA, USA).

COS-7 cells were cultured in RPMI 1640 medium (Sigma–Aldrich) which was supplemented with 10% heat-inactivated FBS (Thermo Fisher Scientific), 2 mM of L-glutamine, 100 U/mL of penicillin, and 100 μg/mL of streptomycin (Thermo Fisher Scientific) at 37°C and 5% CO_2_. The cells were transfected with purified plasmids using Lipofectamine 3000 Reagent (Thermo Fisher Scientific) and cultivated for 48 h after transfection. The cellular localization of EqPD-1-EGFP and EqPD-L1-EGFP was then confirmed using the ZOE Fluorescent Cell Imager (Bio-Rad, Hercules, CA, USA).

### Expression and purification of soluble equine PD-1 and PD-L1 proteins

Soluble forms of EqPD-1 and EqPD-L1 proteins fused with rabbit IgG Fc region (EqPD-1-Ig and EqPD-L1-Ig) were obtained by amplifying cDNAs encoding the extracellular domain fragments of EqPD-1 and EqPD-L1 with signal sequences by PCR with gene-specific primers with restriction enzyme cleavage sites (S1 Table). The amplicons were subcloned into the multicloning site of pCXN2.1(+) (kindly provided by Dr. T. Yokomizo, Juntendo University, Japan) [33] with the gene cassette encoding the Fc region of rabbit IgG. Transient cell lines expressing EqPD-1-Ig and EqPD-L1-Ig were established using an Expi293 Expression System (Thermo Fisher Scientific). Expi293F cells were transfected with pCXN2.1(+)-EqPD-1-Ig and pCXN2.1(+)-EqPD-L1-Ig using Expifectamine (Thermo Fisher Scientific) and cultivated by shaking them in Expi293 medium (Thermo Fisher Scientific) at 37°C and 125 rpm with 8% CO_2_ for 7 days.

Purification of EqPD-1-Ig and EqPD-L1-Ig from the culture supernatants was achieved by affinity chromatography with an Ab-Capcher ExTra (ProteNova, Kagawa, Japan). The buffer was exchanged with PBS by size exclusion chromatography using a PD-10 Desalting Column (GE Healthcare). The purity of EqPD-1-Ig and EqPD-L1-Ig was confirmed by sodium dodecyl sulfate-polyacrylamide gel electrophoresis (SDS-PAGE) in reducing or nonreducing conditions using SuperSep Ace 5%–20% gradient polyacrylamide gel (FUJIFILM Wako Pure Chemical, Osaka, Japan) and 2 × Laemmli Sample Buffer (Bio-Rad). Precision Plus Protein All Blue Standard (Bio-Rad) was used as a molecular-weight size marker. The proteins were visualized with Quick-CBB (FUJIFILM Wako Pure Chemical), and protein concentrations were measured by ultraviolet absorbance at 280 nm with a NanoDrop 8000 Spectrophotometer (Thermo Fisher Scientific).

### Binding assay of EqPD-1 and EqPD-L1

Binding of EqPD-1-Ig and EqPD-L1-Ig to COS-7 cells expressing EqPD-L1-EGFP and EqPD-1-EGFP was investigated using flow cytometry. EqPD-L1-EGFP cells or EqPD-1-EGFP cells were incubated with 10 μg/mL of biotinylated EqPD-1-Ig or EqPD-L1-Ig, respectively, at 37°C for 30 min. Biotinylated rabbit control IgG (Southern Biotech, Birmingham, AL, USA) was used as a negative control. EqPD-1-Ig, EqPD-L1-Ig, and rabbit control IgG were biotinylated using a Lightning-Link Rapid Type A Biotin Conjugation Kit (Innova Biosciences, Cambridge, UK). Cells were washed with PBS containing 1% bovine serum albumin (BSA; Sigma–Aldrich) and labeled using APC-conjugated streptavidin (BioLegend, San Diego, CA, USA) at 25°C for 30 min. After rewashing, cells were immediately analyzed by FACS Verse (BD Biosciences, San Jose, CA, USA).

### Cross-reactivity assay of anti-bovine PD-L1 mAbs against EqPD-L1

EqPD-L1-EGFP cells were incubated with four clones of anti-bovine PD-L1 mAbs (4G12-C1, rat IgG_2a_; 5A2-A1, rat IgG_1_; 6C11-3A11, rat IgG_2a_; 6G7-E1, rat IgM) [19, 34] at 25°C for 20 min to analyze the binding ability of anti-bovine PD-L1 mAbs to EqPD-L1. Rat IgG_1_ (R3-34, BD Biosciences, San Jose, CA, USA), rat IgG_2a_ (R35-95, BD Biosciences), and rat IgM isotype controls (R4-22, BD Biosciences) were used for negative control staining. Cells were washed with 1% BSA-PBS and labeled with APC-conjugated goat anti-rat immunoglobulin antibody (Southern Biotech) at 25°C for 20 min. After rewashing, cells were immediately analyzed by FACS Verse (BD Biosciences).

Fresh and stimulated equine PBMCs were analyzed by flow cytometry to analyze the binding ability of anti-bovine PD-L1 mAbs to equine immune cells. Equine PBMCs (4 × 10^6^ cells) were stimulated in cultivation with 20 ng/mL of PMA (Sigma–Aldrich) and 1 μg/mL of ionomycin (Sigma–Aldrich) for 24 h, as described above. Fresh and stimulated PBMCs were incubated with PBS supplemented with 10% goat serum (Thermo Fisher Scientific) at room temperature for 15 min to prevent nonspecific reactions. Cells were washed and stained with anti-PD-L1 mAbs (5A2-A1, rat IgG_1_; 6C11-3A11; rat IgG_2a_) [19, 34] at room temperature for 30 min. Rat IgG_1_ (R3-34, BD Biosciences) and rat IgG_2a_ isotype controls (R35-95, BD Biosciences) were used for negative control staining. Cells were washed with PBS containing 1% BSA (Sigma–Aldrich) and labeled with APC-conjugated anti-rat Ig antibody (Southern Biotech) at room temperature for 30 min. After rewashing, cells were immediately analyzed by FACS Verse (BD Biosciences).

### Blockade assay of EqPD-1/EqPD-L1 interaction

Blocking assays were conducted in microplates using EqPD-1-Ig and EqPD-L1-Ig to analyze the ability of the anti-PD-L1 mAbs to block PD-1/PD-L1 binding. MaxiSorp Immuno Plates (Thermo Fisher Scientific) were coated with EqPD-1-Ig (1 μg/mL) in carbonate–bicarbonate buffer (Sigma–Aldrich) and blocked using SuperBlock T20 (PBS) Blocking Buffer (Thermo Fisher Scientific). Biotinylated EqPD-L1-Ig was preincubated with anti-PD-L1 mAb 5A2-A1 (rat IgG_1_) [19], 6C11-3A11 (rat IgG_2a_) [34], rat IgG_1_ isotype control (R3-34, BD Biosciences), or rat IgG_2a_ isotype control (R35-95, BD Biosciences) at various concentrations (0, 1.25, 2.5, 5.0, 7.5, and 10 μg/mL) at 37°C for 30 min. The preincubated reagents were added to the microplates and incubated at 37°C for another 30 min. EqPD-L1-Ig binding was detected using horseradish peroxidase-conjugated Neutravidin (Thermo Fisher Scientific) and TMB One Component Substrate (Bethyl Laboratories, Montgomery, TX, USA). Optical density at 450 nm was measured by a microplate reader MTP-900 (Corona Electric, Hitachinaka, Japan). Three independent experiments were each performed in duplicates.

### Immunohistochemical assay of PD-L1

Tumor tissues from four horses bearing EMM were immunohistochemically stained (S2 Table). The tissues were fixed in formalin, embedded into paraffin wax and cut into 4-μm-thick sections. The dried sections were deparaffinized in xylene and hydrated through graded alcohols. Melanin was bleached from the sections using 0.25% potassium permanganate and 2% oxalic acid. Antigen retrieval was achieved using 0.01 M citrate buffer (pH 6.0) by microwave heating. Endogenous peroxidase activity was blocked by incubating the sections in methanol containing 0.3% hydrogen peroxide. The sections were incubated with or without anti-PD-L1 mAb (6C11-3A11, rat IgG_2a_) [34] at 4°C overnight, followed by detection using Vectastain Elite ABC Rat IgG kit (Vector Laboratories, Burlingame, CA, USA). The immunoreaction was visualized using 3,3’-diaminobenzidine tetrahydrochloride. All immunostained sections were examined under an optical microscope.

### Immunoactivation assay using equine PBMCs

To determine the effects of inhibiting the PD-1/PD-L1 interaction on equine immune cells, equine PBMCs were cultured with 10 μg/mL of anti-PD-L1 mAb (6C11-3A11, rat IgG_2a_) [34] or rat IgG_2a_ control (Bio X Cell, West Lebanon, NH, USA) in the presence of 0.1 μg/mL of Staphylococcal enterotoxin B (SEB; Sigma–Aldrich) at 37°C with 5% CO_2_ for 3 days. All cell cultures were grown in 96-well round-bottomed plates (Corning Inc., Corning, NY, USA) containing 4 × 10^5^ PBMCs in 200 μl of RPMI 1640 medium (Sigma–Aldrich) which was supplemented with 10% heat-inactivated FBS, 2 mM of L-glutamine, 100 U/mL of penicillin, and 100 μg/mL of streptomycin (Thermo Fisher Scientific). Cytokine concentrations in the culture supernatants were determined using an Equine IFN-γ ELISA Development Kit (Mabtech, Nacka Strand, Sweden) and an Equine IL-2 DuoSet ELISA Development Kit (R&D Systems, Minneapolis, MN, USA). Measurements were performed in duplicates in accordance with the manufacturer’s protocol.

### Statistical analysis

Significant differences were identified using Wilcoxon signed-rank test or Tukey’s test. All statistical tests were performed using the MEPHAS (http://www.gen-info.osaka-u.ac.jp/MEPHAS/) statistical analysis program. Statistical significance was set as *p* < 0.05.

## Results

### Molecular cloning and sequence analysis of equine PD-1/PD-L1

**Fig 1:**
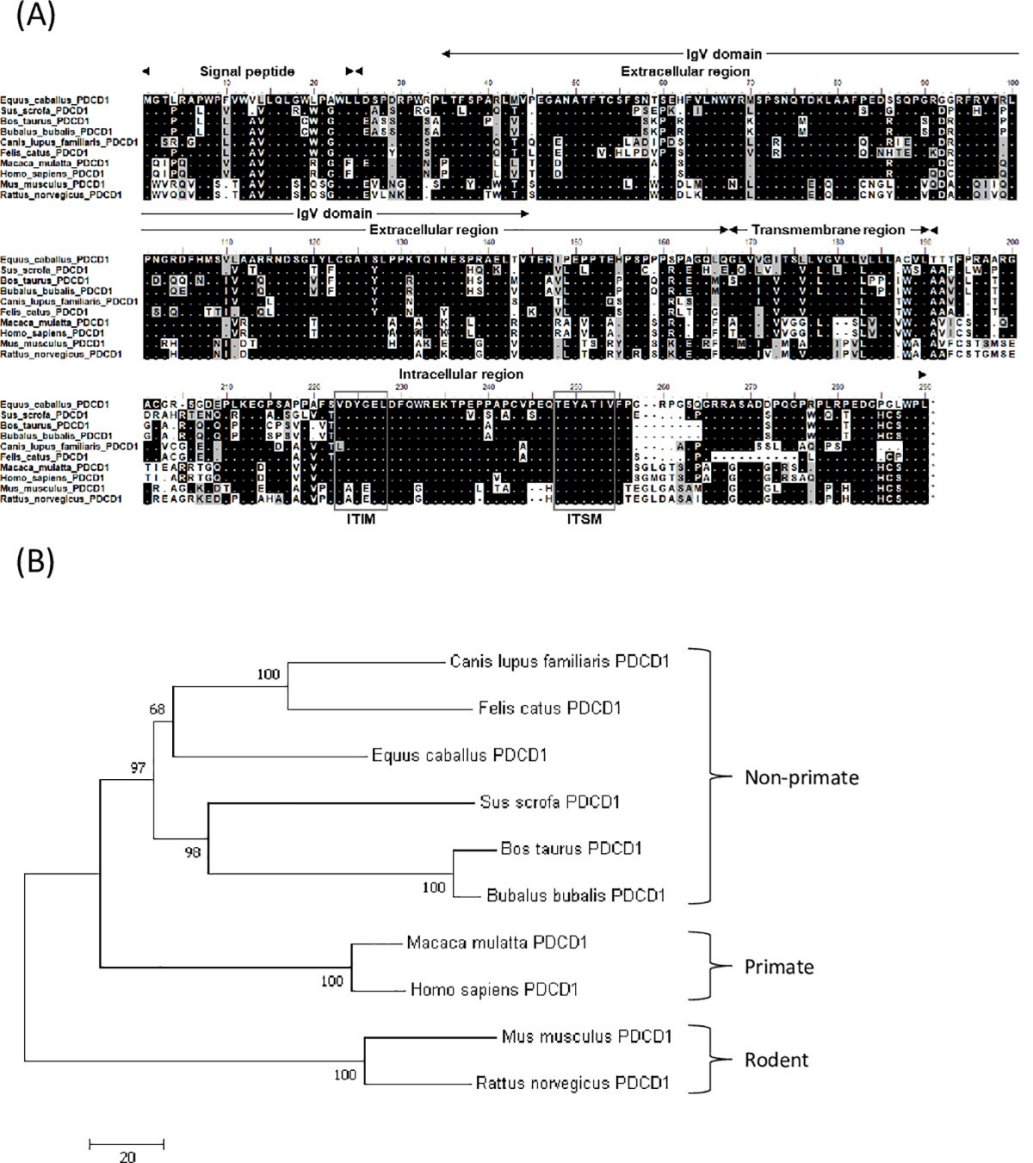
Sequence analysis of EqPD-1. (A) Multiple alignment of amino acid sequences of equine and vertebrate PD-1. Predicted domains and motifs of EqPD-1 are shown. EqPD-1 consists of a signal peptide, an extracellular region, a transmembrane region, and an intracellular region. The cytoplasmic tail of PD-1 contains the ITIM and ITSM motifs. (B) Phylogenetic tree of EqPD-1 sequence in relation to those of other vertebrate species. The bootstrap consensus tree was inferred from 1000 replicates with the neighbor-joining method using the MEGA 7.0 software. The scale indicates the divergence time. The GenBank accession numbers of nucleotide sequences used in these analyses are listed in S3 Table.

**Fig 2:**
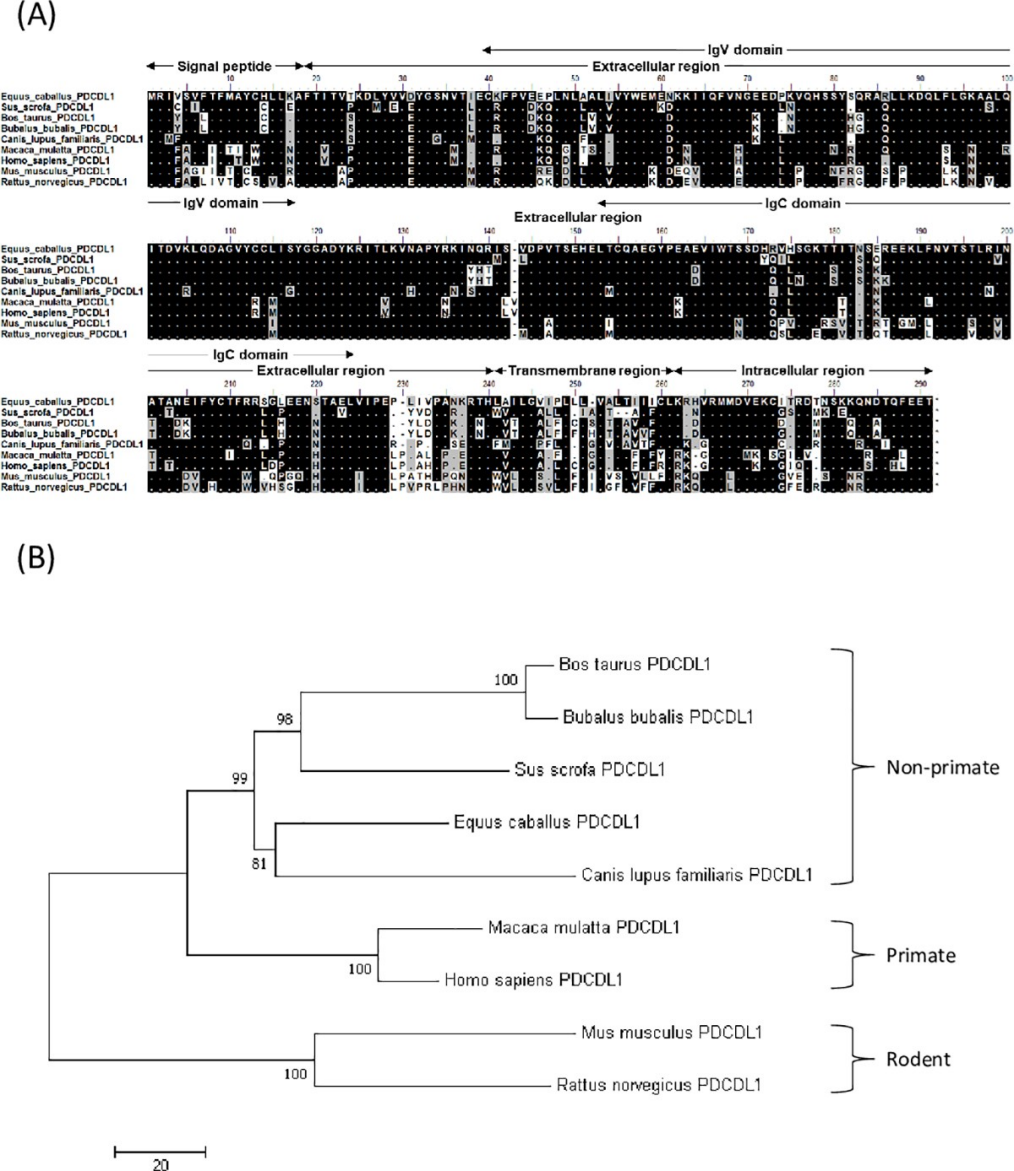
Sequence analysis of EqPD-L1. (A) Multiple alignment of PD-L1 amino acid sequences of equine and vertebrate PD-L1. Predicted domains and motifs of EqPD-L1 are shown in the figure. EqPD-L1 consists of a signal peptide, an extracellular region, a transmembrane region, and an intracellular region. (B) Phylogenetic tree of the EqPD-L1 sequence in relation to other vertebrate species. The bootstrap consensus tree was inferred from 1000 replicates with the neighbor-joining method using the MEGA 7.0 software. The scale indicates the divergence time. The GenBank accession numbers for the nucleotide sequences used in these analyses are listed in S3 Table.

Firstly, we identified cDNA sequences encoding EqPD-1 and EqPD-L1. Figs 1A and 2A show the putative amino acid sequences of EqPD-1 and EqPD-L1, respectively. EqPD-1 and EqPD-L1 consist of a putative signal peptide, an extracellular region, a transmembrane region, and an intracellular region. These were expected to be type I transmembrane proteins as orthologues in other species. A conserved domain search identified an immunoglobulin variable (IgV)-like domain in the extracellular regions of EqPD-1. IgV-like and immunoglobulin constant (IgC)-like domains were observed in the extracellular regions of EqPD-L1. The intracellular region of EqPD-1 contained two structural motifs, an immunoreceptor tyrosine-based inhibitory motif (ITIM) and an immunoreceptor tyrosine-based switch motif (ITSM). Phylogenetic analyses revealed that EqPD-1 and EqPD-L1 were clustered into the non-primate group (Figs 1B and 2B). EqPD-1 had 70.4%, 69.0%, 75.6%, 69.2%, and 58.7% amino acid similarities to pig, cattle, dog, human, and mouse respectively (Table 1). EqPD-L1 had 81.5%, 80.9%, 83.7%, 79.0%, and 67.9% amino acid similarities to pig, cattle, dog, human, and mouse, respectively (Table 2).

**Table 1:** Similarities of nucleotide and amino acid sequences of PD-1 among mammalian species.

	Horse	Pig	Cattle	Water buffalo	Dog	Cat	Human	Monkey	Mouse	Rat
Horse	-	82.5	79.1	79.3	83.3	80.5	79.7	80.3	72.4	73.1
Pig	70.4	-	80.2	80.8	78.5	78.5	76.8	76.7	69.6	70.8
Cattle	69.0	71.5	-	97.5	78.3	78.3	74.5	74.7	68.5	67.8
Water buffalo	70.8	73.2	96.8	-	78.8	78.6	74.5	75.2	69.5	68.4
Dog	75.6	67.9	69.3	71.0	-	88.3	76.7	77.2	70.4	71.5
Cat	72.2	65.2	69.7	71.1	82.2	-	76.4	75.9	69.8	70.8
Human	69.2	63.7	63.7	63.4	65.3	62.4	-	95.8	71.7	72.5
Monkey	70.6	64.1	65.1	65.8	65.3	62.0	95.8	-	71.6	72.5
Mouse	58.7	55.1	51.3	53.1	56.5	51.7	59.3	60.0	-	91.3
Rat	61.5	58.3	56.8	59.0	58.2	54.1	58.9	61.3	85.7	-

Upper section; similarities (%) in nucleotide level, lower section; similarities (%) in amino acid level.

**Table 2:** Similarities of nucleotide and amino acid sequences of PD-L1 among mammalian species.

	Horse	Pig	Cattle	Water buffalo	Dog	Human	Monkey	Mouse	Rat
Horse	-	88.8	87.8	87.3	88.1	86.6	85.2	75.6	75.7
Pig	81.5	-	88.1	88.2	85.1	84.3	83.3	74.4	75.5
Cattle	80.9	81.3	-	98.2	84.0	83.0	81.8	73.8	75.2
Water buffalo	79.9	80.6	96.5	-	83.7	82.3	81.5	74.2	75.7
Dog	83.7	76.8	78.5	77.8	-	83.2	82.1	73.4	74.5
Human	79.0	73.3	73.1	72.0	75.9	-	95.7	76.3	76.4
Monkey	76.6	71.6	72.7	72.0	74.2	91.3	-	75.2	75.3
Mouse	67.9	67.4	65.1	66.2	67.0	69.4	68.3	-	87.3
Rat	68.3	67.4	67.3	67.6	68.1	69.7	68.3	83.4	-

Upper section; similarities (%) in nucleotide level, lower section; similarities (%) in amino acid level.

### Interaction of EqPD-1 and EqPD-L1

**Fig 3:**
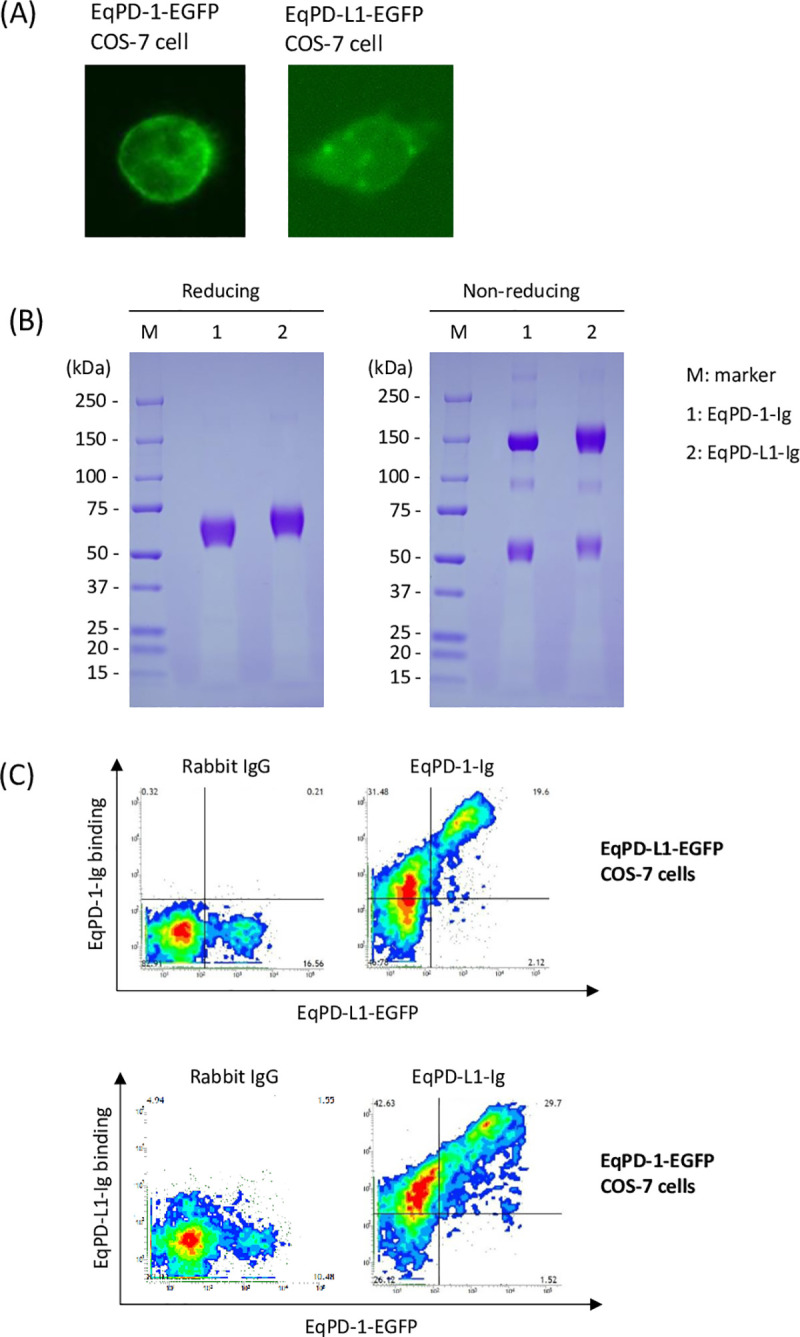
Establishment of EqPD-1- or EqPD-L1-expressing cells and Ig fusion soluble proteins. (A) EqPD-1-EGFP or EqPD-L1-EGFP-expressing COS-7 cell. The subcellular distributions of EqPD-1-EGFP and EqPD-L1-EGFP in transfected COS-7 cells were analyzed using a fluorescence microscope. (B) Production and purification of Ig fusion EqPD-1 and EqPD-L1 proteins. EqPD-1-Ig and EqPD-L1-Ig were purified from the culture supernatant and analyzed using SDS-PAGE. The original uncropped and unadjusted image of the SDS-PAGE analysis is provided as S1 Fig. (C) Interaction of EqPD-1 and EqPD-L1. EqPD-1-EGFP or EqPD-L1-expressing COS-7 cells were incubated with EqPD-L1-Ig or EqPD-1-Ig, respectively. The binding of the Ig fusion proteins was labeled using Alexa Flour 647 conjugated anti-rabbit IgG antibody and analyzed by flow cytometry.

We evaluated the cellular localization of EqPD-1-EGFP and EqPD-L1-EGFP proteins in the overexpressed COS-7 cell lines and found them to be localized on the cell surface (Fig 3A). We developed soluble recombinant EqPD-1-Ig and EqPD-L1-Ig in the Expi293 Expression System to analyze interactions of EqPD-1 and EqPD-L1 proteins. EqPD-1-Ig and EqPD-L1-Ig were successfully purified from culture supernatants and confirmed to be dimerized by disulfide bonds in the hinge region of rabbit IgG (Fig 3B). Predicted molecular weights of EqPD-1-Ig and EqPD-L1-Ig are 42.4 kDa and 50.3 kDa under reduced condition and 84.8 kDa and 100.6 kDa under non-reduced condition, respectively. These proteins would be modified by post-translational modifications, such as glycosylation, and have larger molecular weights than predicted.

We used flow cytometry to analyze the interactions of EqPD-1-Ig or EqPD-L1-Ig with EqPD-L1-EGFP- or EqPD-1-EGFP-expressing cells, respectively. This revealed that EqPD-1-Ig binding to EqPD-L1-EGFP-expressing cells depends on the expression level of EqPD-1-EGFP (Fig 3C). We also confirmed that EqPD-L1-Ig binds to EqPD-1-EGFP-expressing cells in an expression-dependent manner (Fig 3C).

### Cross-reactivity of anti-bovine PD-L1 mAbs against EqPD-L1

**Fig 4:**
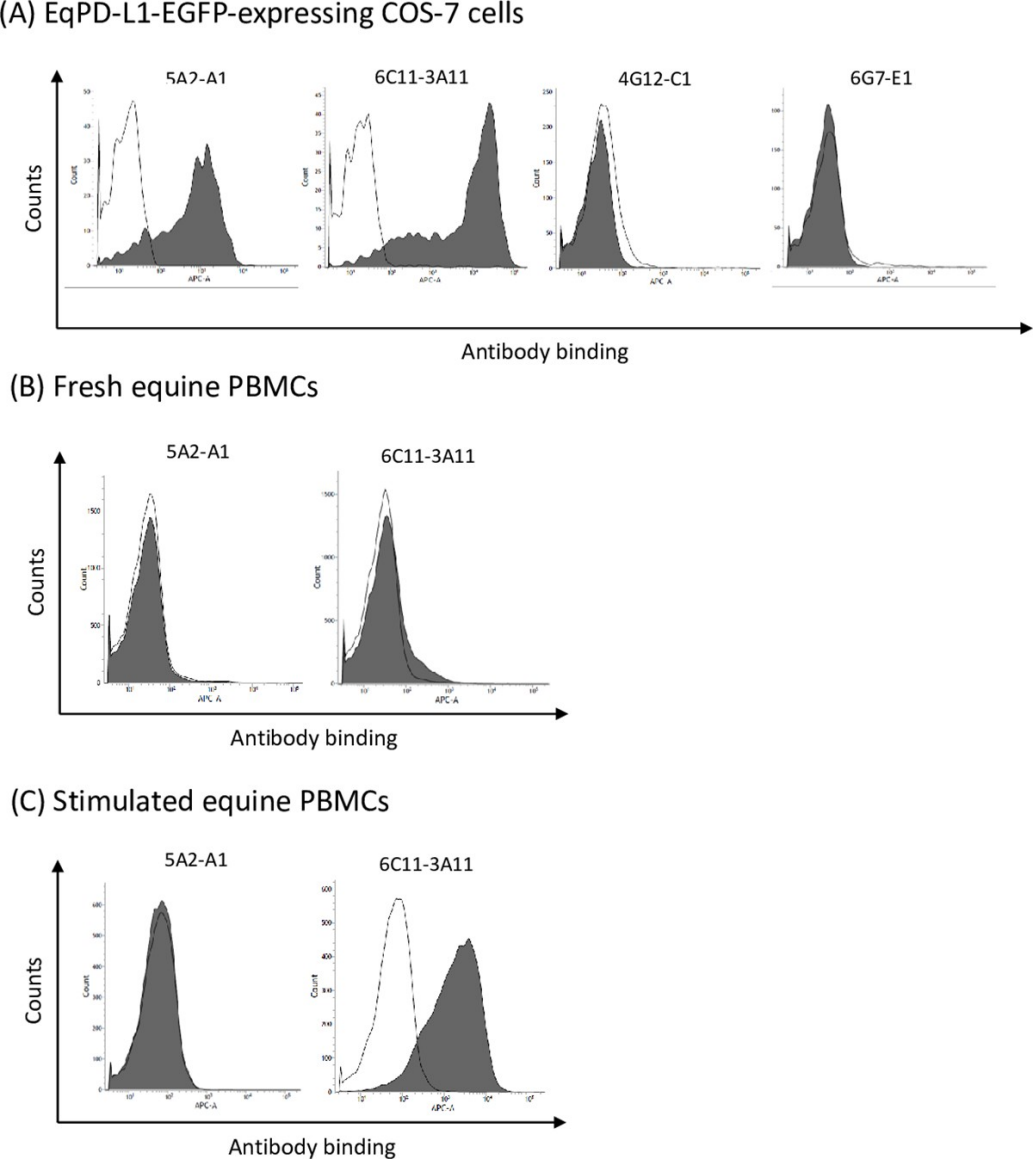
Cross-reactivity of anti-bovine PD-L1 mAbs against EqPD-L1. (A–C) Binding activities of anti-bovine PD-L1 mAbs (5A2-A1, 6C11-3A11, 4G12-C1, and 6G7-E1) to (A) EqPD-L1-EGFP-expressing COS-7 cells, (B) fresh equine PBMCs, and (C) equine PBMCs stimulated with PMA and ionomycin for 24 h. The binding of the primary mAbs was labeled with APC-conjugated anti-rat Ig antibody and analyzed by flow cytometry. Rat IgG_1_ and IgG_2a_ controls were used as isotype-matched negative controls.

We evaluated cross-reactivity of our previously established anti-bovine PD-L1 mAbs [5, 20] against EqPD-L1 and found that two out of the four tested mAbs (5A2-A1 and 6C11-3A11) detected EqPD-L1-EGFP overexpressed on COS-7 cells (Fig 4A). Of all the tested mAbs, 6C11-3A11 showed the strongest binding to EqPD-L1-EGFP-expressing cells (Fig 4A). We also tested the reactivity of 5A2-A1 and 6C11-3A11 mAbs against fresh and stimulated equine PBMCs and found that 6C11-3A11 mAb binds to both fresh and stimulated PBMCs (Fig 4B and 4C). PD-L1 expression was upregulated on PBMCs through stimulation with PMA and ionomycin (Fig 4C).

### Inhibition of EqPD-1/EqPD-L1 binding by anti-PD-L1 mAbs

**Fig 5:**
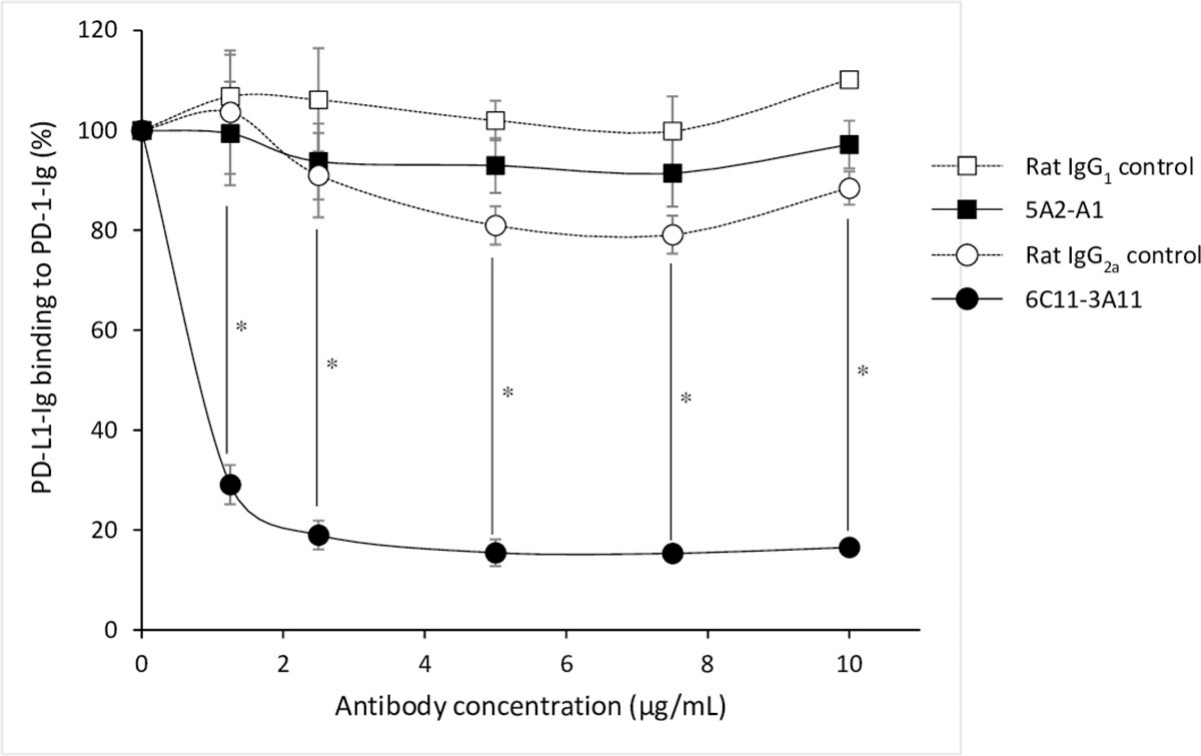
Inhibition of equine PD-1/PD-L1 binding by anti-PD-L1 mAbs. The blocking effect of anti-PD-L1 mAb on the binding of EqPD-L1-Ig to EqPD-1-Ig. EqPD-1-Ig was coated on a microwell plate. Biotinylated EqPD-L1-Ig was preincubated with various concentrations of anti-PD-L1 mAb (5A2-A1 or 6C11-3A11) and then incubated in the coated microwell plate. Rat IgG_1_ and IgG_2a_ controls were used as isotype-matched negative controls. Each curve represents the relative binding of EqPD-L1-Ig preincubated with antibodies compared to that preincubated with no antibody. Each point indicates the average value of three independent experiments. Significant differences between each treatment were identified using Tukey’s test. An asterisk (*) indicates *p* < 0.05. The original data of this assay is provided as S2 Fig.

We used ELISA to investigate whether the cross-reactive anti-bovine PD-L1 mAbs interfered with their interaction with EqPD-1/EqPD-L1. Although 6C11-3A11 mAb, blocked the binding of EqPD-L1-Ig with EqPD-1-Ig in a dose-dependent manner, but 5A2-A1 mAb did not (Fig 5).

### Immunohistochemical analysis of PD-L1 in EMM

**Fig 6:**
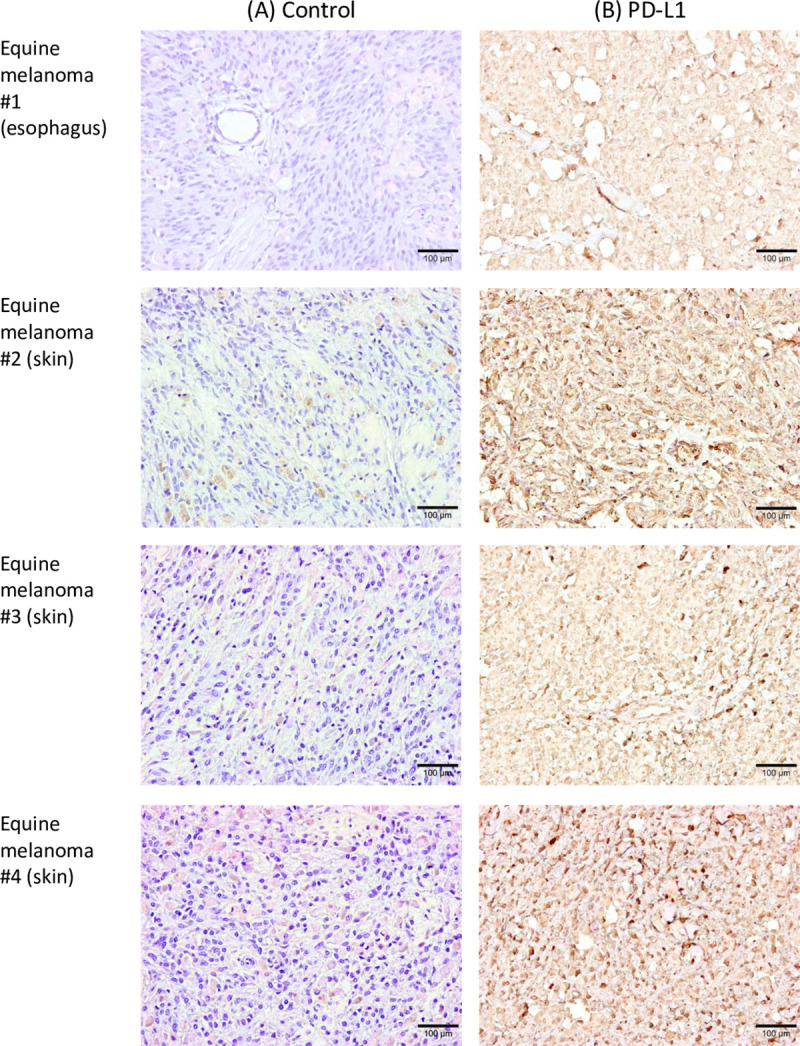
Immunohistochemical analysis of PD-L1 in EMM. Immunohistochemical staining of PD-L1 in tumor tissues of horses with melanoma (#1–#4). Each section was stained (A) without a primary antibody (control) or (B) using anti-bovine PD-L1 mAb (6C11-3A11). Further information of tumor specimens is shown in S2 Table.

PD-L1 has been shown to be upregulated in many types of tumors in dogs and humans [25, 26, 35]. Among canine malignant cancers, malignant melanoma has the highest positive rates for PD-L1 expression [26]. Gray horses are susceptible to melanoma and approximately 80% of them develop EMM in their lifetimes [17]. We hypothesized that PD-L1 plays a role in the development of EMM. Therefore, we analyzed the expression of PD-L1 in tumor tissues of EMM by immunohistochemistry. PD-L1 was detected in all EMM samples (*n* = 4, Fig 6B).

### Immune activation in equine PBMCs by anti-PD-L1 mAb

**Fig 7:**
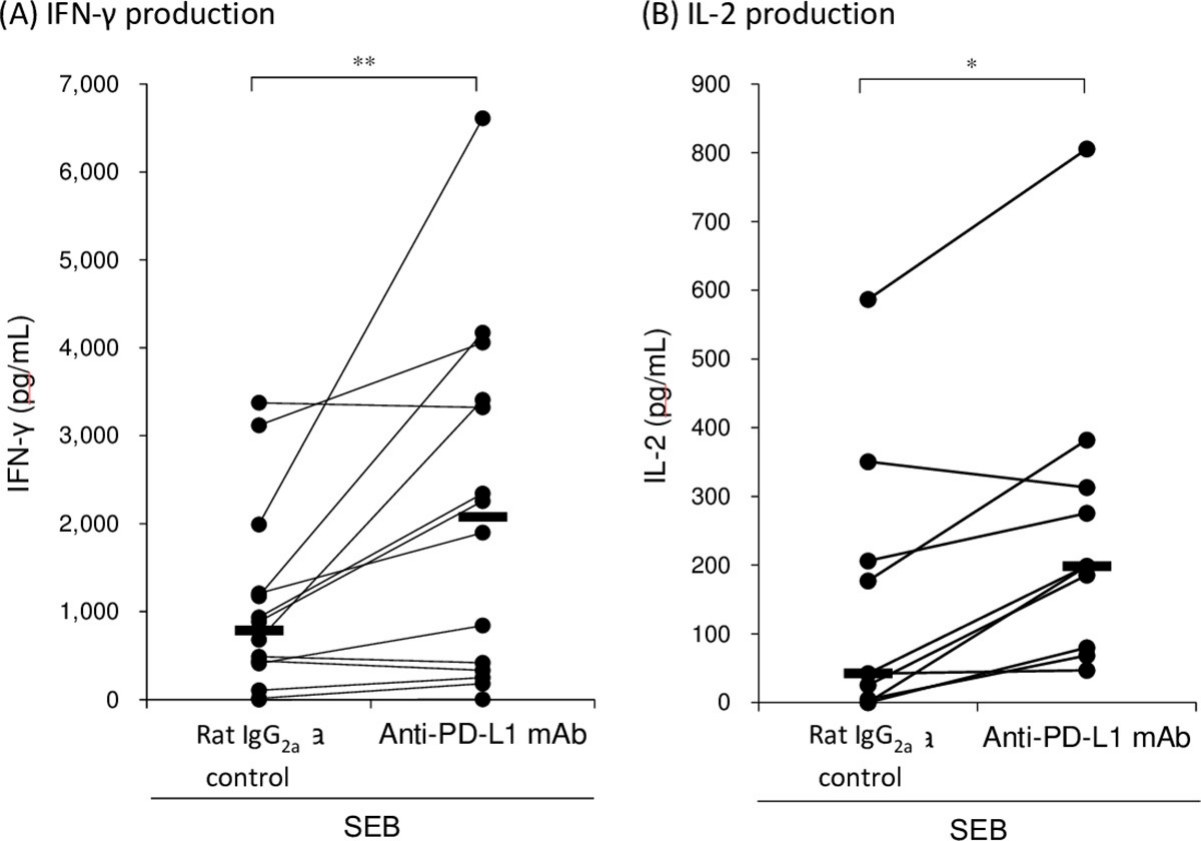
Effect of PD-L1 blockade on IFN-γ and IL-2 production. PBMCs isolated from healthy horses were cultured with anti-PD-L1 mAb (6C11-3A11) or rat IgG_2a_ control in the presence of SEB. The culture supernatants were harvested 3 days later and IFN-γ and IL-2 concentrations were measured by ELISA (IFN-γ: *n* = 14 and IL-2: *n* = 9). Significant differences between each treatment were identified using Wilcoxon signed-rank test. Asterisks (* and **) indicate *p* < 0.05 and < 0.01, respectively. The original data of this assay is provided as S3 Fig.

We analyzed immune activation effects by PD-1/PD-L1 inhibition in the PBMC culture assays using anti-PD-L1 blocking mAb, 6C11-3A11. We found that PD-L1 blockade by the mAb 6C11-3A11 significantly induced IFN-γ production by equine PBMCs under stimulation with SEB (Fig 7A). Additionally, production of IL-2 was increased by PD-L1 inhibition (Fig 7B). These results indicate that PD-1/PD-L1 blockade enhanced Th1 cytokine production in equine PBMCs, suggesting that the anti-PD-L1 blocking antibody has an application as an immunomodulatory agent for horses.

## Discussion

The greater longevity of the horse population has increased the risks of chronic diseases, such as laminitis, pituitary pars intermedia dysfunction, recurrent airway obstruction, osteoarthritis, and neoplasia, and increased multimorbidity in horses [36, 37]. However, treatments available for chronic diseases in horses, including malignant tumors, are few. Therefore, new treatment options are being sought.

Malignant melanoma is one of the most common cutaneous neoplasia in horses [38]. Surgical treatment is successful in the early stages of the disease, but it is not feasible in cases with multiple tumor burdens and metastases. Although a number of systemic treatments have been tested, no effective systemic therapy is currently available for EMM. To overcome the current situation, novel therapeutic strategies, including immunotherapy, are warranted for EMM.

A variety of immunotherapies have been developed and tested in clinical trials to treat tumors in humans, and immune checkpoint inhibitors such as anti-PD-1 and anti-PD-L1 antibodies are currently used with notable success for the treatment of multiple human cancers [12–15]. Blockade therapy using anti-PD-L1 antibody resulted in long-term tumor regression and prolonged progression-free survival in advanced melanoma in humans [12]. Based on these advancements in human medicine, immune checkpoint inhibitors may reasonably be expected to yield equally promising results in the treatment of EMM [18]. However, no studies have been conducted on the PD-1/PD-L1 pathway in horses as yet.

Our recent research revealed that the PD-1/PD-L1 pathway plays a critical role in immune exhaustion and disease progression in bovine chronic infections and canine malignant cancers [20–26]. Moreover, we established anti-PD-L1 and anti-PD-1 blocking antibodies for therapeutic application in cattle and dogs [27, 28, 31]. Clinical studies have confirmed the antiviral, antibacterial, and antitumoral effects of antibody treatments [27–31]. However, the blockade effect of the PD-1/PD-L1 pathway had not been tested in horses. Hence, we aimed to identify cDNA sequences of EqPD-1 and EqPD-L1 and evaluate the function of our anti-bovine PD-L1 mAbs using *in vitro* assays.

We found that one of the anti-bovine PD-L1 mAbs (6C11-3A11) strongly recognized EqPD-L1, blocked the interaction of EqPD-1/EqPD-L1, and enhanced the Th1 cytokine response *in vitro*. In contrast, the other cross-reactive mAb (5A2-A1) bound to the EqPD-L1-overexpressing cell line, but not to equine PBMCs, failed to block the interaction of EqPD-1/EqPD-L1. The difference between the results using these two mAbs would depend on the epitope and binding affinity of the mAbs. The expression level of PD-L1 could be the highest in the overexpressing cells, followed in order by stimulated and fresh PBMCs. The binding affinity of 5A2-A1 would not be enough to detect PD-L1 on equine PBMCs. The mAb 6C11-3A11 may be used to aid investigation into the expression and immunological function of PD-L1 in future horse studies. Additionally, we discovered that PD-L1 is expressed in EMM tumor tissues. Further studies are required to analyze expression of PD-L1 in other horse tumors and chronic diseases.

The mechanism of PD-L1 upregulation during EMM progression has yet to be elucidated. Generally, PD-L1 expression is regulated by a substantial number of mediators including inflammatory cytokine signaling, oncogenic signaling, microRNAs, genetic alteration of the PD-L1 locus, and post-translational regulators [39]. In gray horses, a gene duplication in intron 6 of *STX17* (synataxin 17) contributes to a *cis*-acting regulatory mutation resulting in a very high incidence of EMM [40]. This gene duplication induces constitutive activation of the extracellular signal-regulated kinase (ERK) pathway and tumorigenesis in EMM [41, 42]. The MEK-ERK signaling pathway regulates PD-L1 gene expression via crosstalk with inflammatory cytokine signaling including the IFN-γ-STAT1 pathway [43–45]. Hence, the regulatory mechanism of PD-L1 expression in gray horses merits investigation as a natural model of tumorigenesis.

Our results indicate that the PD-1/PD-L1 pathway offers a potential target for immunotherapy against EMM. In future immunotherapy applications, blocking antibodies should be engineered into suitable forms for administration to horses. Chimeric antibodies, for instance, may facilitate clinical trial research into the clinical efficacy of anti-PD-L1 antibody in the treatment of EMM. Further research is required to develop this novel immunotherapy strategy in horses.

## Supporting information

S1 Fig The original uncropped and unadjusted image of the SDS-PAGE gel, related to Fig 3B. (PPTX)

S2 Fig The original data of blockade assay of EqPD-1/EqPD-L1 interaction, related to Fig 5. (A–C) Optimal density at 450nm (OD450) and relative value of the EqPD-1-Ig/EqPD-L1-Ig binding in three independent experiments. (D) Average of the relative values of the EqPD-1-Ig/EqPD-L1-Ig.
(PPTX)

S3 Fig The original data of immunoactivation assay using equine PBMCs, related to Fig 7. (A) IFN-γ (*n* = 14) and (B) IL-2 production (*n* = 9) from equine PBMCs cultured with anti-PD-L1 mAb (6C11-3A11) or rat IgG2a control in the presence of SEB.
(PPTX)

S1 Table Primer sequences used in this study. (PPTX)

S2 Table Samples of EMM used in the immunohistochemical analysis. (PPTX)

S3 Table GenBank accession number of the nucleotide sequences used in this study. (PPTX)
