# Supplementary material for: PD-L1 expression in equine malignant melanoma and functional effects of PD-L1 blockade
Source: PLoS One. 2020 Nov 20;15(11):e0234218. doi: 10.1371/journal.pone.0234218 (PMC7678989; doi:10.1371/journal.pone.0234218)
Supplement: S1 Fig — (PPTX) [file pone.0234218.s001.pptx]

## Slide 1
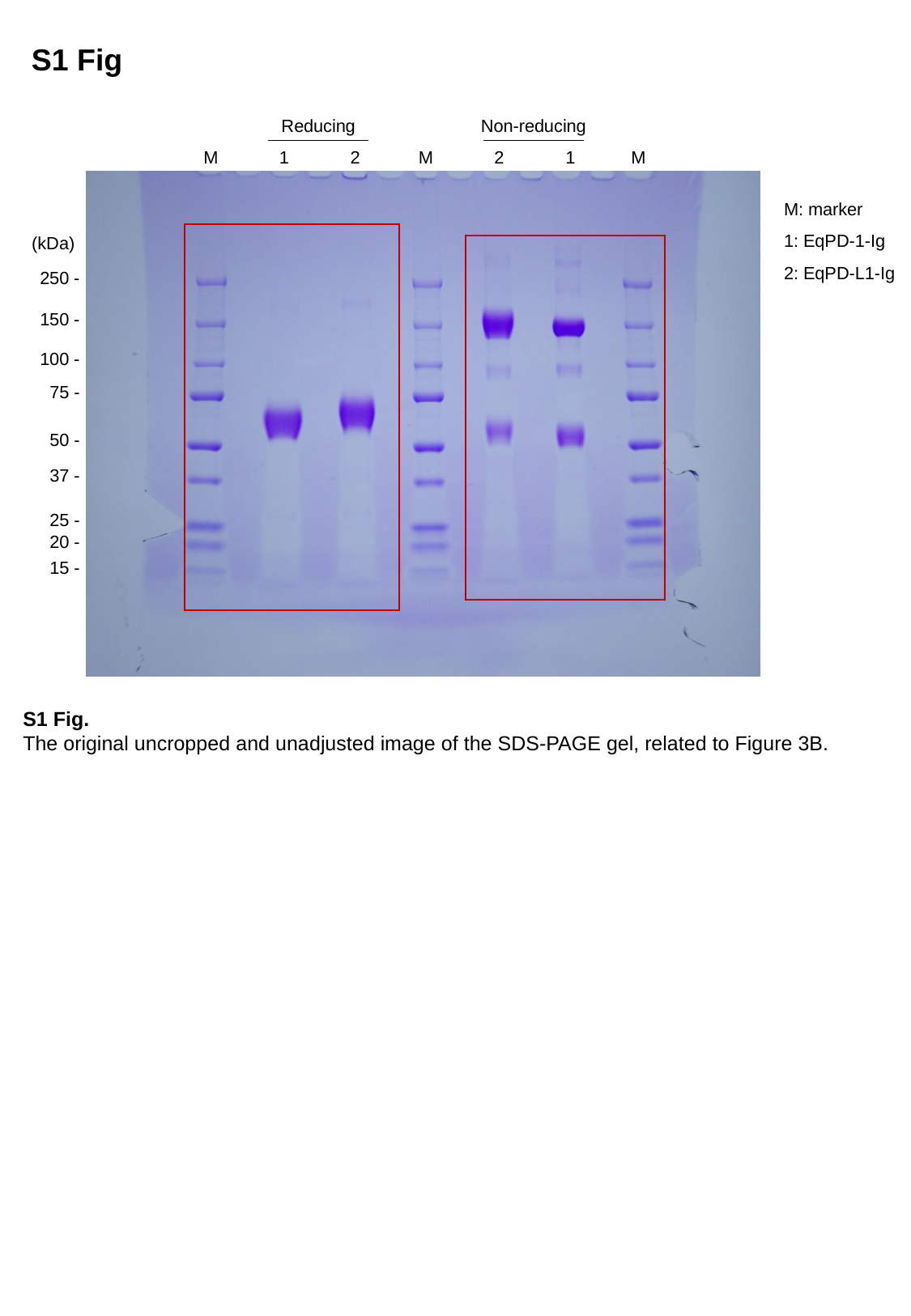

S1 Fig
Reducing
Non-reducing
M
1
2
M
2
1
M
M: marker
1: EqPD-1-Ig
2: EqPD-L1-Ig
(kDa)
250 -
150 -
100 -
75 -
50 -
37 -
25 -
20 -
15 -
S1 Fig.
The original uncropped and unadjusted image of the SDS-PAGE gel, related to Figure 3B.
