# Supplementary material for: PD-L1 expression in equine malignant melanoma and functional effects of PD-L1 blockade
Source: PLoS One. 2020 Nov 20;15(11):e0234218. doi: 10.1371/journal.pone.0234218 (PMC7678989; doi:10.1371/journal.pone.0234218)
Supplement: S2 Fig — (A–C) Optimal density at 450nm (OD450) and relative value of the EqPD-1-Ig/EqPD-L1-Ig binding in three independent experiments. (D) Average of the relative values of the EqPD-1-Ig/EqPD-L1-Ig. (PPTX) [file pone.0234218.s002.pptx]

## Slide 1
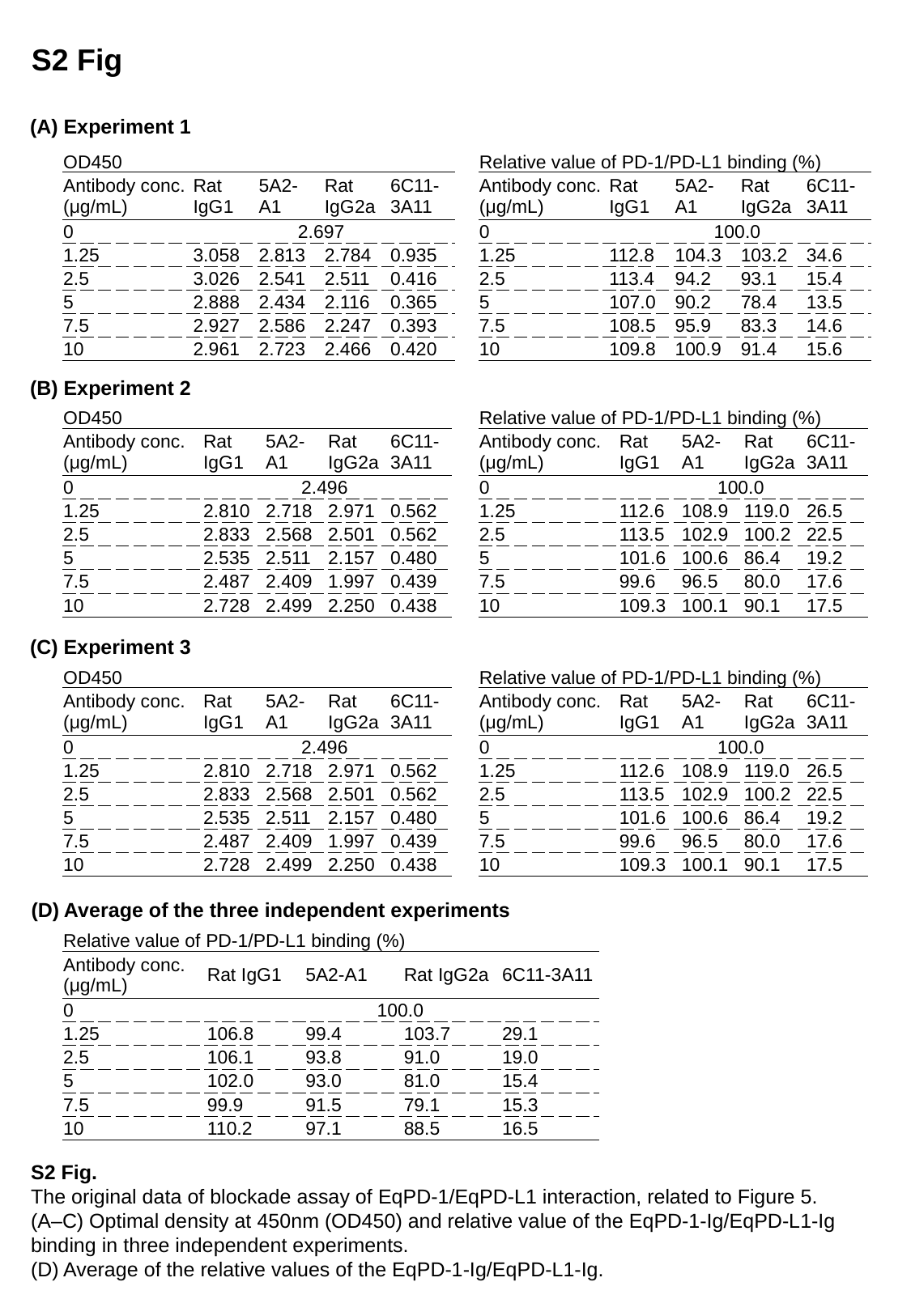

S2 Fig
(A) Experiment 1
| OD450 | | | | |
| --- | --- | --- | --- | --- |
| Antibody conc. (μg/mL) | Rat IgG1 | 5A2- A1 | Rat IgG2a | 6C11- 3A11 |
| 0 | 2.697 | | | |
| 1.25 | 3.058 | 2.813 | 2.784 | 0.935 |
| 2.5 | 3.026 | 2.541 | 2.511 | 0.416 |
| 5 | 2.888 | 2.434 | 2.116 | 0.365 |
| 7.5 | 2.927 | 2.586 | 2.247 | 0.393 |
| 10 | 2.961 | 2.723 | 2.466 | 0.420 |
| Relative value of PD-1/PD-L1 binding (%) | | | | |
| --- | --- | --- | --- | --- |
| Antibody conc. (μg/mL) | Rat IgG1 | 5A2- A1 | Rat IgG2a | 6C11- 3A11 |
| 0 | 100.0 | | | |
| 1.25 | 112.8 | 104.3 | 103.2 | 34.6 |
| 2.5 | 113.4 | 94.2 | 93.1 | 15.4 |
| 5 | 107.0 | 90.2 | 78.4 | 13.5 |
| 7.5 | 108.5 | 95.9 | 83.3 | 14.6 |
| 10 | 109.8 | 100.9 | 91.4 | 15.6 |
(B) Experiment 2
| OD450 | | | | |
| --- | --- | --- | --- | --- |
| Antibody conc. (μg/mL) | Rat IgG1 | 5A2- A1 | Rat IgG2a | 6C11- 3A11 |
| 0 | 2.496 | | | |
| 1.25 | 2.810 | 2.718 | 2.971 | 0.562 |
| 2.5 | 2.833 | 2.568 | 2.501 | 0.562 |
| 5 | 2.535 | 2.511 | 2.157 | 0.480 |
| 7.5 | 2.487 | 2.409 | 1.997 | 0.439 |
| 10 | 2.728 | 2.499 | 2.250 | 0.438 |
| Relative value of PD-1/PD-L1 binding (%) | | | | |
| --- | --- | --- | --- | --- |
| Antibody conc. (μg/mL) | Rat IgG1 | 5A2- A1 | Rat IgG2a | 6C11- 3A11 |
| 0 | 100.0 | | | |
| 1.25 | 112.6 | 108.9 | 119.0 | 26.5 |
| 2.5 | 113.5 | 102.9 | 100.2 | 22.5 |
| 5 | 101.6 | 100.6 | 86.4 | 19.2 |
| 7.5 | 99.6 | 96.5 | 80.0 | 17.6 |
| 10 | 109.3 | 100.1 | 90.1 | 17.5 |
(C) Experiment 3
| OD450 | | | | |
| --- | --- | --- | --- | --- |
| Antibody conc. (μg/mL) | Rat IgG1 | 5A2- A1 | Rat IgG2a | 6C11- 3A11 |
| 0 | 2.496 | | | |
| 1.25 | 2.810 | 2.718 | 2.971 | 0.562 |
| 2.5 | 2.833 | 2.568 | 2.501 | 0.562 |
| 5 | 2.535 | 2.511 | 2.157 | 0.480 |
| 7.5 | 2.487 | 2.409 | 1.997 | 0.439 |
| 10 | 2.728 | 2.499 | 2.250 | 0.438 |
| Relative value of PD-1/PD-L1 binding (%) | | | | |
| --- | --- | --- | --- | --- |
| Antibody conc. (μg/mL) | Rat IgG1 | 5A2- A1 | Rat IgG2a | 6C11- 3A11 |
| 0 | 100.0 | | | |
| 1.25 | 112.6 | 108.9 | 119.0 | 26.5 |
| 2.5 | 113.5 | 102.9 | 100.2 | 22.5 |
| 5 | 101.6 | 100.6 | 86.4 | 19.2 |
| 7.5 | 99.6 | 96.5 | 80.0 | 17.6 |
| 10 | 109.3 | 100.1 | 90.1 | 17.5 |
(D) Average of the three independent experiments
| Relative value of PD-1/PD-L1 binding (%) | | | | |
| --- | --- | --- | --- | --- |
| Antibody conc. (μg/mL) | Rat IgG1 | 5A2-A1 | Rat IgG2a | 6C11-3A11 |
| 0 | 100.0 | | | |
| 1.25 | 106.8 | 99.4 | 103.7 | 29.1 |
| 2.5 | 106.1 | 93.8 | 91.0 | 19.0 |
| 5 | 102.0 | 93.0 | 81.0 | 15.4 |
| 7.5 | 99.9 | 91.5 | 79.1 | 15.3 |
| 10 | 110.2 | 97.1 | 88.5 | 16.5 |
S2 Fig.
The original data of blockade assay of EqPD-1/EqPD-L1 interaction, related to Figure 5.
(A–C) Optimal density at 450nm (OD450) and relative value of the EqPD-1-Ig/EqPD-L1-Ig binding in three independent experiments.
(D) Average of the relative values of the EqPD-1-Ig/EqPD-L1-Ig.
