# Supplementary material for: PD-L1 expression in equine malignant melanoma and functional effects of PD-L1 blockade
Source: PLoS One. 2020 Nov 20;15(11):e0234218. doi: 10.1371/journal.pone.0234218 (PMC7678989; doi:10.1371/journal.pone.0234218)
Supplement: S3 Fig — (A) IFN-γ (n = 14) and (B) IL-2 production (n = 9) from equine PBMCs cultured with anti-PD-L1 mAb (6C11-3A11) or rat IgG2a control in the presence of SEB. (PPTX) [file pone.0234218.s003.pptx]

## Slide 1
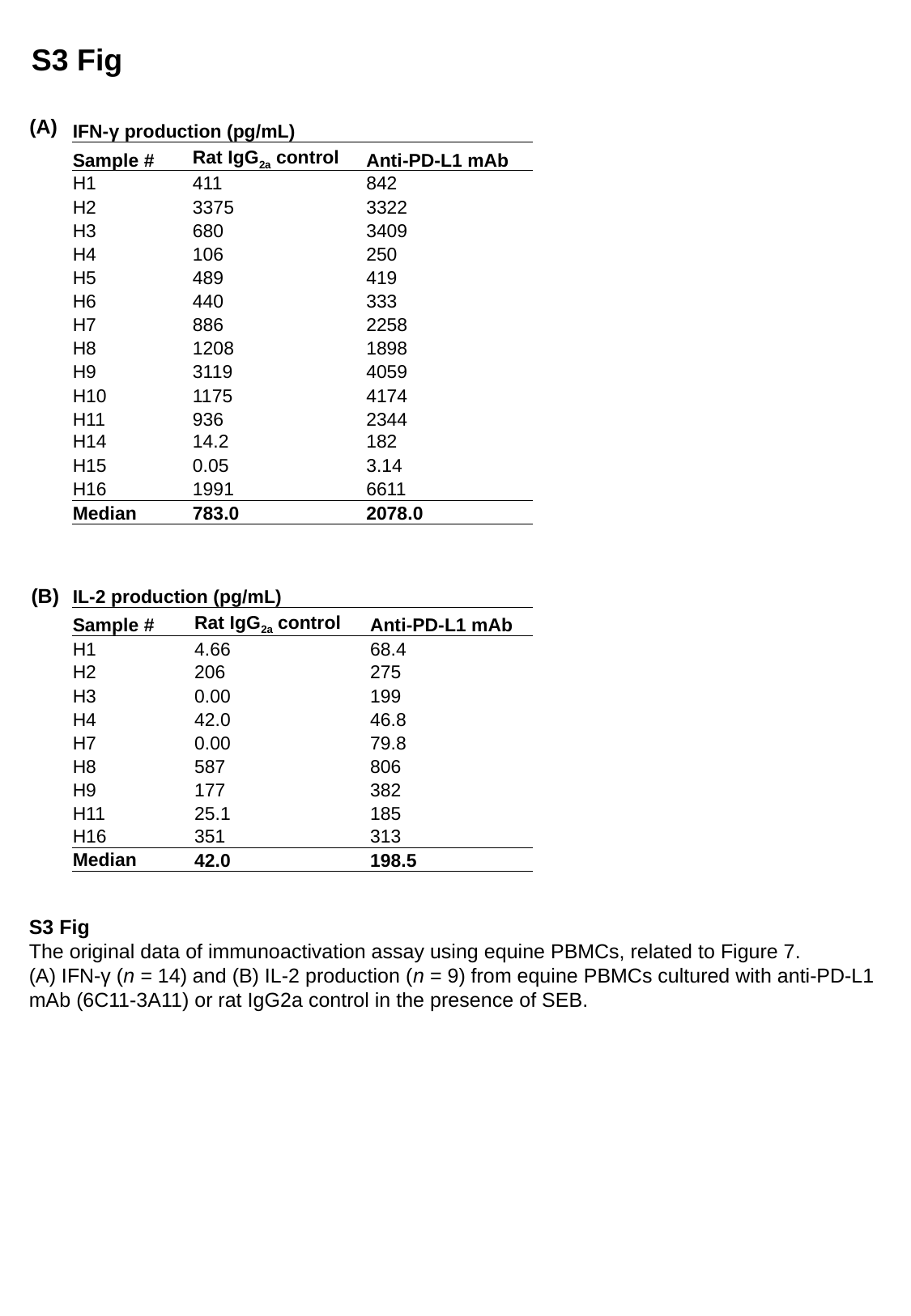

S3 Fig
(A)
| IFN-γ production (pg/mL) | | |
| --- | --- | --- |
| Sample # | Rat IgG2a control | Anti-PD-L1 mAb |
| H1 | 411 | 842 |
| H2 | 3375 | 3322 |
| H3 | 680 | 3409 |
| H4 | 106 | 250 |
| H5 | 489 | 419 |
| H6 | 440 | 333 |
| H7 | 886 | 2258 |
| H8 | 1208 | 1898 |
| H9 | 3119 | 4059 |
| H10 | 1175 | 4174 |
| H11 | 936 | 2344 |
| H14 | 14.2 | 182 |
| H15 | 0.05 | 3.14 |
| H16 | 1991 | 6611 |
| Median | 783.0 | 2078.0 |
(B)
| IL-2 production (pg/mL) | | |
| --- | --- | --- |
| Sample # | Rat IgG2a control | Anti-PD-L1 mAb |
| H1 | 4.66 | 68.4 |
| H2 | 206 | 275 |
| H3 | 0.00 | 199 |
| H4 | 42.0 | 46.8 |
| H7 | 0.00 | 79.8 |
| H8 | 587 | 806 |
| H9 | 177 | 382 |
| H11 | 25.1 | 185 |
| H16 | 351 | 313 |
| Median | 42.0 | 198.5 |
S3 Fig
The original data of immunoactivation assay using equine PBMCs, related to Figure 7.
(A) IFN-γ (n = 14) and (B) IL-2 production (n = 9) from equine PBMCs cultured with anti-PD-L1 mAb (6C11-3A11) or rat IgG2a control in the presence of SEB.
