# Supplementary material for: PD-L1 expression in equine malignant melanoma and functional effects of PD-L1 blockade
Source: PLoS One. 2020 Nov 20;15(11):e0234218. doi: 10.1371/journal.pone.0234218 (PMC7678989; doi:10.1371/journal.pone.0234218)
Supplement: S1 Table — (PPTX) [file pone.0234218.s004.pptx]

## Slide 1
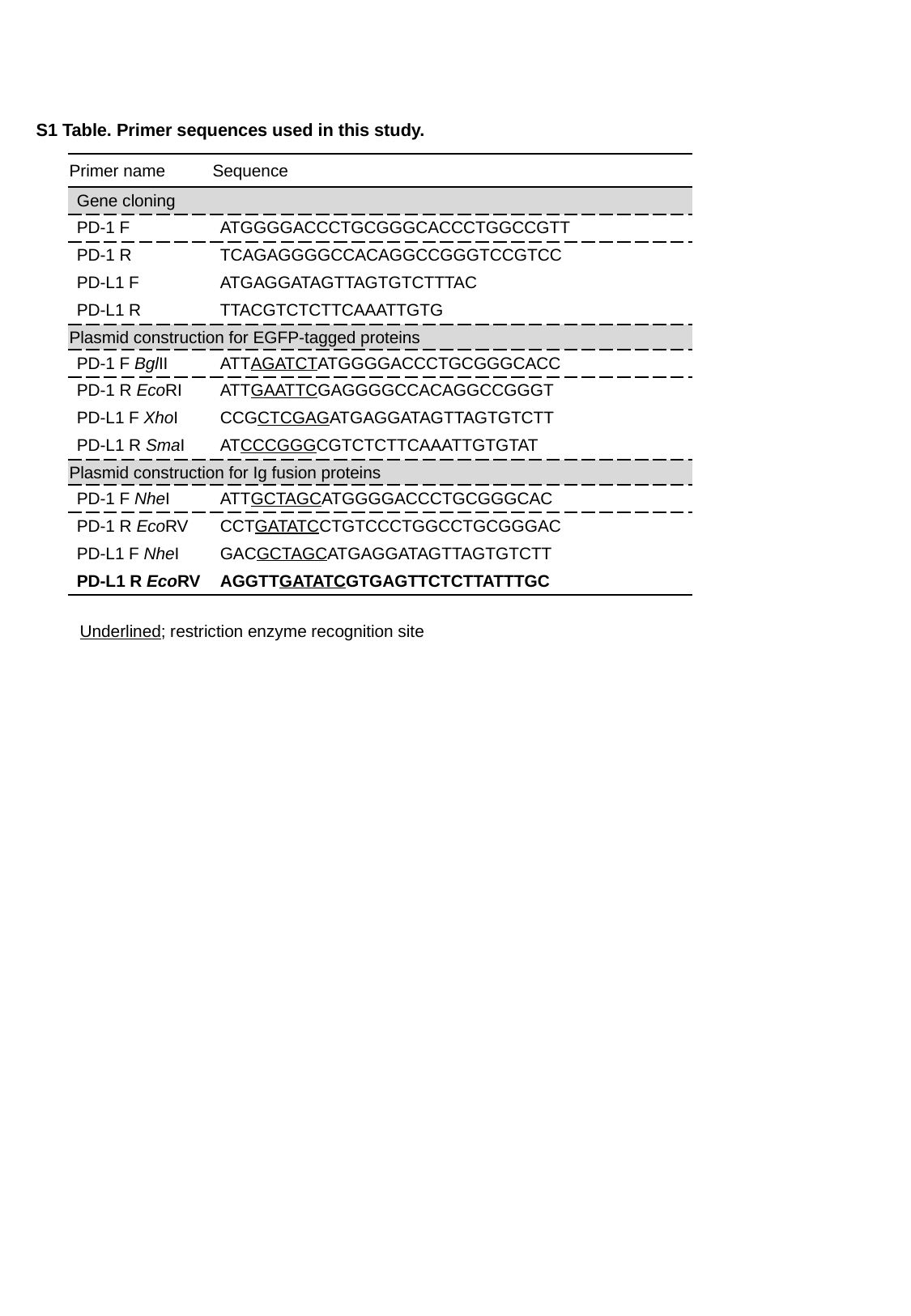

S1 Table. Primer sequences used in this study.
| Primer name | Sequence |
| --- | --- |
| Gene cloning | |
| PD-1 F | ATGGGGACCCTGCGGGCACCCTGGCCGTT |
| PD-1 R | TCAGAGGGGCCACAGGCCGGGTCCGTCC |
| PD-L1 F | ATGAGGATAGTTAGTGTCTTTAC |
| PD-L1 R | TTACGTCTCTTCAAATTGTG |
| Plasmid construction for EGFP-tagged proteins | |
| PD-1 F BglII | ATTAGATCTATGGGGACCCTGCGGGCACC |
| PD-1 R EcoRI | ATTGAATTCGAGGGGCCACAGGCCGGGT |
| PD-L1 F XhoI | CCGCTCGAGATGAGGATAGTTAGTGTCTT |
| PD-L1 R SmaI | ATCCCGGGCGTCTCTTCAAATTGTGTAT |
| Plasmid construction for Ig fusion proteins | |
| PD-1 F NheI | ATTGCTAGCATGGGGACCCTGCGGGCAC |
| PD-1 R EcoRV | CCTGATATCCTGTCCCTGGCCTGCGGGAC |
| PD-L1 F NheI | GACGCTAGCATGAGGATAGTTAGTGTCTT |
| PD-L1 R EcoRV | AGGTTGATATCGTGAGTTCTCTTATTTGC |
Underlined; restriction enzyme recognition site
