# Supplementary material for: PD-L1 expression in equine malignant melanoma and functional effects of PD-L1 blockade
Source: PLoS One. 2020 Nov 20;15(11):e0234218. doi: 10.1371/journal.pone.0234218 (PMC7678989; doi:10.1371/journal.pone.0234218)
Supplement: S2 Table — (PPTX) [file pone.0234218.s005.pptx]

## Slide 1
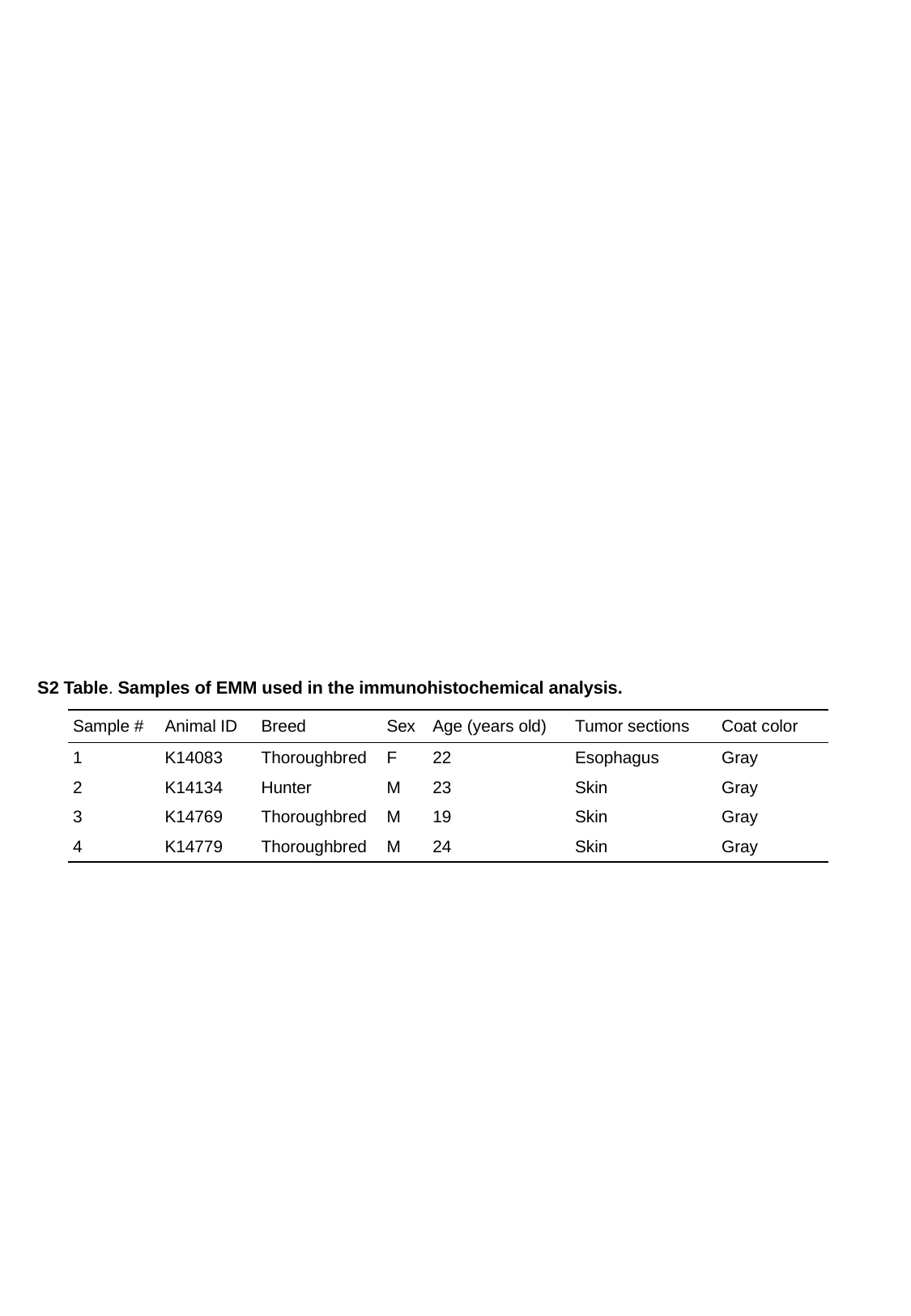

S2 Table. Samples of EMM used in the immunohistochemical analysis.
| Sample # | Animal ID | Breed | Sex | Age (years old) | Tumor sections | Coat color |
| --- | --- | --- | --- | --- | --- | --- |
| 1 | K14083 | Thoroughbred | F | 22 | Esophagus | Gray |
| 2 | K14134 | Hunter | M | 23 | Skin | Gray |
| 3 | K14769 | Thoroughbred | M | 19 | Skin | Gray |
| 4 | K14779 | Thoroughbred | M | 24 | Skin | Gray |
