# Supplementary material for: PD-L1 expression in equine malignant melanoma and functional effects of PD-L1 blockade
Source: PLoS One. 2020 Nov 20;15(11):e0234218. doi: 10.1371/journal.pone.0234218 (PMC7678989; doi:10.1371/journal.pone.0234218)
Supplement: S3 Table — (PPTX) [file pone.0234218.s006.pptx]

## Slide 1
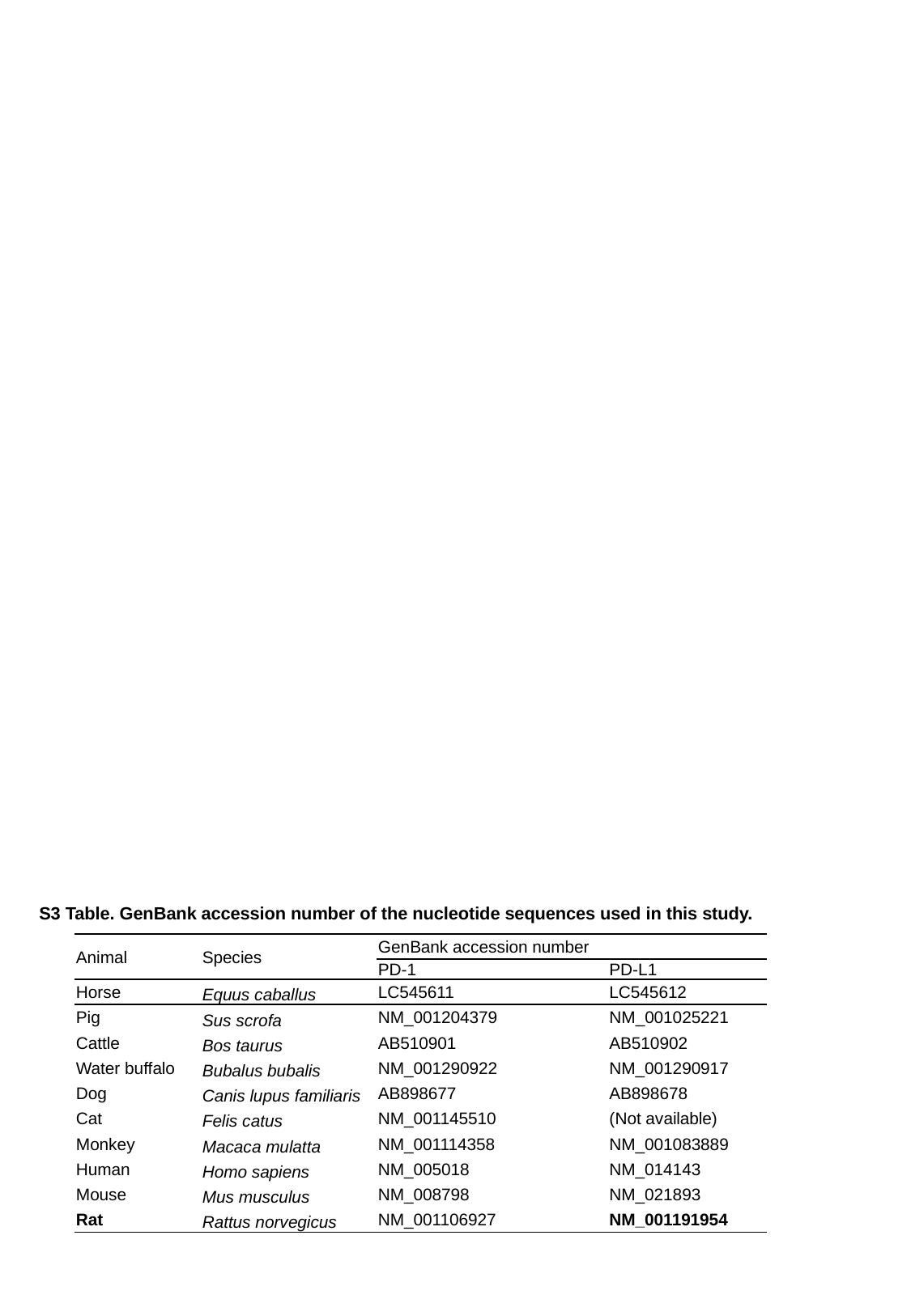

S3 Table. GenBank accession number of the nucleotide sequences used in this study.
| Animal | Species | GenBank accession number | |
| --- | --- | --- | --- |
| | | PD-1 | PD-L1 |
| Horse | Equus caballus | LC545611 | LC545612 |
| Pig | Sus scrofa | NM\_001204379 | NM\_001025221 |
| Cattle | Bos taurus | AB510901 | AB510902 |
| Water buffalo | Bubalus bubalis | NM\_001290922 | NM\_001290917 |
| Dog | Canis lupus familiaris | AB898677 | AB898678 |
| Cat | Felis catus | NM\_001145510 | (Not available) |
| Monkey | Macaca mulatta | NM\_001114358 | NM\_001083889 |
| Human | Homo sapiens | NM\_005018 | NM\_014143 |
| Mouse | Mus musculus | NM\_008798 | NM\_021893 |
| Rat | Rattus norvegicus | NM\_001106927 | NM\_001191954 |
